# Investigating exploration for deep reinforcement learning of concentric tube robot control

**DOI:** 10.1007/s11548-020-02194-z

**Published:** 2020-06-06

**Authors:** Keshav Iyengar, George Dwyer, Danail Stoyanov

**Affiliations:** Charles Bell House, 43-45 Foley St, Fitzrovia, London, W1W 7TY UK

**Keywords:** Deep reinforcement learning, Concentric tube robots, Robot control, Surgical robotics

## Abstract

**Purpose:**

Concentric tube robots are composed of multiple concentric, pre-curved, super-elastic, telescopic tubes that are compliant and have a small diameter suitable for interventions that must be minimally invasive like fetal surgery. Combinations of rotation and extension of the tubes can alter the robot’s shape but the inverse kinematics are complex to model due to the challenge of incorporating friction and other tube interactions or manufacturing imperfections. We propose a model-free reinforcement learning approach to form the inverse kinematics solution and directly obtain a control policy.

**Method:**

Three exploration strategies are shown for deep deterministic policy gradient with hindsight experience replay for concentric tube robots in simulation environments. The aim is to overcome the joint to Cartesian sampling bias and be scalable with the number of robotic tubes. To compare strategies, evaluation of the trained policy network to selected Cartesian goals and associated errors are analyzed. The learned control policy is demonstrated with trajectory following tasks.

**Results:**

Separation of extension and rotation joints for Gaussian exploration is required to overcome Cartesian sampling bias. Parameter noise and Ornstein–Uhlenbeck were found to be optimal strategies with less than 1 mm error in all simulation environments. Various trajectories can be followed with the optimal exploration strategy learned policy at high joint extension values. Our inverse kinematics solver in evaluation has 0.44 mm extension and $0.3^{\circ }$ rotation error.

**Conclusion:**

We demonstrate the feasibility of effective model-free control for concentric tube robots. Directly using the control policy, arbitrary trajectories can be followed and this is an important step towards overcoming the challenge of concentric tube robot control for clinical use in minimally invasive interventions.

## Introduction

**Fig. 1:**
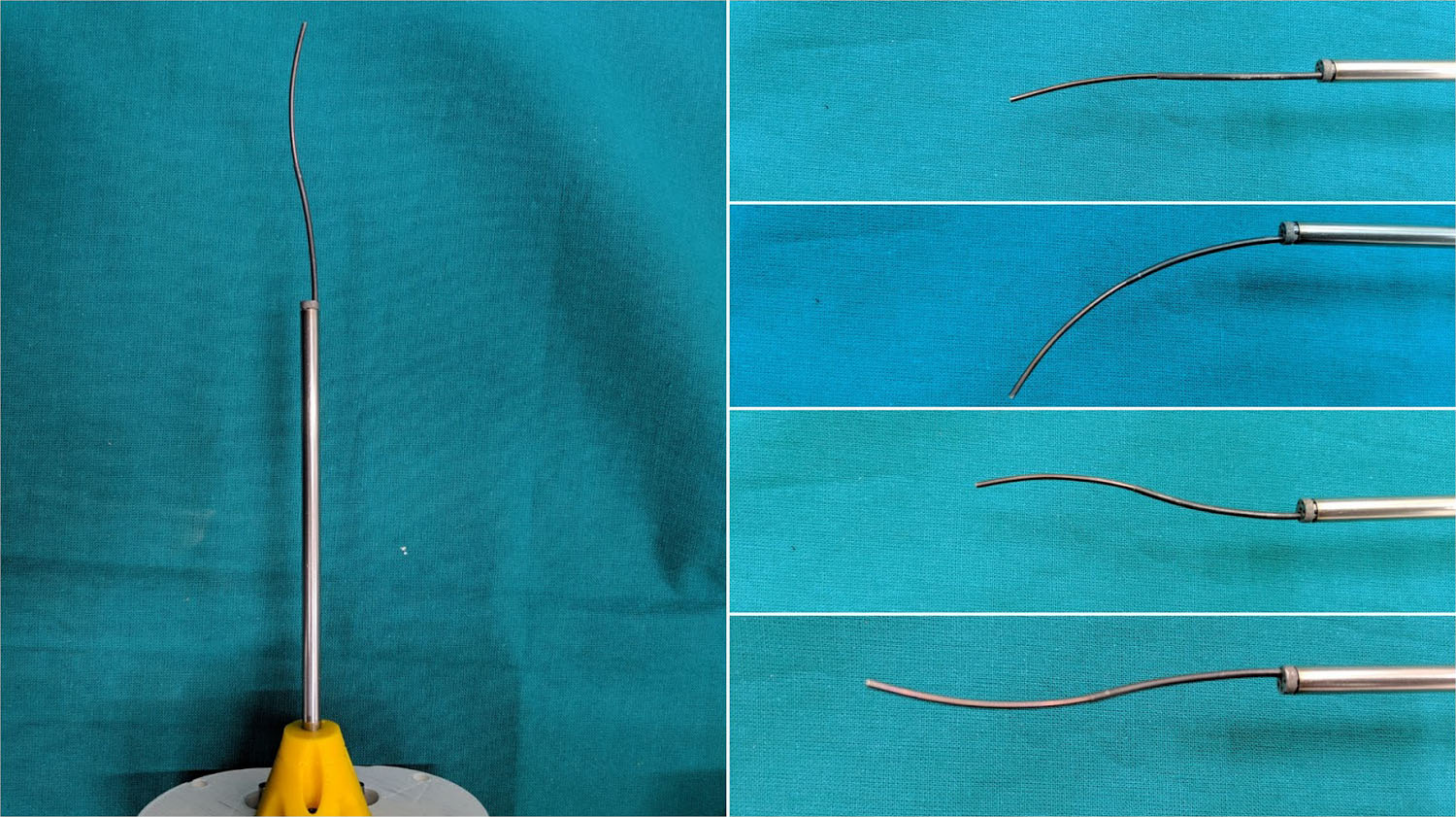
The figure shows the curvilinear path of a two tube concentric tube robot designed for fetal surgery [6]. The series of images on the right illustrate the workspace of the robotic instrument as the system extends

Robotic articulation can enable minimally invasive surgery (MIS) for challenging procedures where minimally invasive approaches are typically prohibited by manual straight instrumentation. Fetal surgery for the treatment of congenital malformations in the fetus is one such specialization [6, 7]. While various robotic systems and architectures have been proposed for fetal interventions, one of the most important requirements in instrumentation is to have flexible articulated instruments while maintaining a very small instrument profile to minimize trauma at the entry port. Concentric tube robots [4] are a sub-type of continuum robot that use neighbouring tube interactions of bending and twisting when rotated and translated to form curvilinear paths as shown in Fig. 1. These paths can avoid anatomical structures, be compliant and still offer dexterity at the tip, and importantly for fetal interventions, be implemented at very small diameters. However, concentric tube robot designs have major challenges in achieving reliable control because of robot kinematics modelling error [14]. Common modelling approaches of concentric tube robot kinematics are based on special Coserat rods for each tube undergoing bending and torsion that lead to no analytical solution for robots consisting of two tubes or more or for pre-curvature that varies with length [5, 19]. Additional factors like friction and tube tolerances have been investigated [14] but are difficult to integrate because of the large computational load for modelling. Inverse kinematics strategies applied are common approaches like numerical root finding or differential inverse kinematics [3, 5]. Comparing data-driven approach (DDA) methods to inverse kinematics strategies for a tendon-driven continuum robot showed DDA approaches are faster and more accurate [21]. A model-free DDA method would be beneficial because of accuracy in real scenarios compared to current model-based inverse kinematics strategies. The modelling inconsistencies are manufacturing tolerances, unmodelled tube interactions and unpredictable contacts. Furthermore, unlike neural network approaches that have been proposed [2, 8] reinforcement learning can be trained in successively complex environments and eventually to a real environment by combining training parameters [17]. Reinforcement learning is then data efficient, if the cost of collecting real life data is high.

In this paper, we investigate different exploration strategies in model-free deep reinforcement learning for concentric tube robots. Specifically, zero-mean Gaussian noise, Ornstein–Uhlenbeck noise and parameter noise to enhance DDA control strategies. Joint sampling bias and number of tubes scalability are two main challenges that are encountered with this approach. We show how learning overcomes these challenges and demonstrate that control based on reinforcement learning can also directly follow a trajectory, a feature not available to other DDA methods. Path planners and teleoperation methods can incorporate this model-free solver for fetal surgery for which concentric tube mechanisms are being developed.

## Prior work and preliminaries

Reinforcement learning is a framework to map states to actions by maximizing a numerical reward signal. The reward signal is from an environment and an agent uses this signal to determine future actions. As described in [20], if a system is in state $s_t$ at timestep *t*, and a certain action $a_t$ is taken, then it enters state $s_{t+1}$ and receives a reward signal $r_{t}$. A policy $\pi$ must be developed taking into account the current reward and all subsequent future rewards before episode termination. A policy determines which action is likely to have the greatest cumulative reward over the sequence of all future actions. An estimate of the overall expected reward of the current state-action pair is known as the *Q*-value. A recursive relationship exists between the *Q*-value and policy in the form of the Bellman equation:

1 $$\begin{aligned} Q^\pi (s_t, a_t) = \mathbb {E}_{r_t, s_{t+1} \sim E} \left[ r_t + \gamma Q^\pi (s_{t+1}, \pi (s_{t+1}) )\right] , \end{aligned}$$

**Fig. 2:**
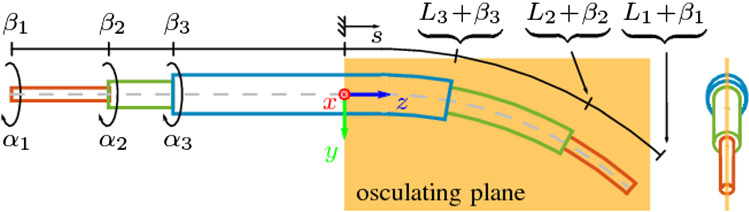
3 tube illustration in a single plane adapted from [8]. Tubes can be rotated ($\alpha _i$) and translated ($\beta _i$) relative to each other. The arc length variable *s* describes the robot shape with its respective tube length $L_i$

where $s_t$ and $a_t$ are the state and action at timestep *t*, $Q^\pi$ is the *Q*-value function following policy $\pi$ and $\gamma$ is the discount factor. The reward $r_t$ and next state $s_{t+1}$ are from the environment *E*. Actor-critic methods of reinforcement learning use a critic to estimate the *Q*-value function and an actor to estimate the policy function and update the policy function in the direction suggested by the critic with policy gradients [13]. Reinforcement learning problems are formulated as a Markov Decision Process (MDP). An MDP consists of set of states, set of actions, a reward function and the discount factor. All states follow the Markov property and transitions between states are fully defined with an action and reward value. In the literature, there are two ways incorporate continuous states and actions. The first is discritizing the state and actions and the second is a black box simulation to simulate state, action and resulting next states with reward [13]. The former often results in the curse of dimensionality as fine control produces a large state and action space. The latter is used extensively and is chosen for this work.

We are not aware of previous work using reinforcement learning for concentric tube robots but two DDA methods exist. One uses simulated data to train a multi-layer perceptron (MLP) network for inverse kinematics of a 3 tube robot with one variable curvature section [2]. The rotation configuration space is split into four quadrants resulting in an output of a single extension joint value per tube and 4 rotation joint values per tube. The correct joint tuple is then selected by examining the least forward kinematics tip error. To avoid bias during training, extension values less than 30% of the maximum extension value are ignored. The simulation accuracy results demonstrate Cartesian error is below 0.8 mm running at 50 Hz in MATLAB. Another approach also uses a MLP framework for inverse kinematics and contributes a novel joint space representation [8] following a trigonometric joint representation [12] with adaptation for concentric tube robots. The work defines a cylindrical form $\gamma _i$, with $i=0$ being the innermost tube and $i=n$ being the outermost tube:

2 $$\begin{aligned} \gamma _i = \{ \gamma _{1,i}, \gamma _{2,i}, \gamma _{3,i} \} = \{ \cos (\alpha _i), \sin (\alpha _i), \beta _i \}, \end{aligned}$$

which describes the *i*th tube as a triplet. The rotatory joint of tube *i*, $\alpha _i$ can be retrieved by

3 $$\begin{aligned} \alpha _i = \text {atan2} \{ \gamma _{2,i}, \gamma _{1,i} \}. \end{aligned}$$

The extension joint of tube *i*, $\beta _i$, can be retrieved directly and has constraints

4 $$\begin{aligned}&0 \ge \beta _n \ge \dots \ge \beta _2 \ge \beta _1, \end{aligned}$$

5 $$\begin{aligned}&0 \le L_n + \beta _n \le \dots \le L_2 + \beta _2 \le L_1 + \beta _1, \end{aligned}$$

where *n* is the number of tubes and $L_i$ is the overall length of tube *i*. The joint variables are visualized in Fig. 2. A recent study [9], investigated various joint space representations and confirmed that the cylindrical representation performs much better for MLP frameworks as compared a simple rotation and extension form. In experimentation, hardware training and evaluation was done with a 3 tube concentric tube robot, the actuation error was 4.0 mm in translation and $8.3^{\circ }$ with 60,000 training samples. The cylindrical form, extension constraints and order of tube indexing is directly used as the joint representation for our reinforcement learning strategy.

A major challenge of model-free reinforcement learning in continuous state and action spaces is exploration [15]. An advantage of using an off-policy algorithm is the learned policy does not have to be the one used for training. During training, the action selected by the agent is perturbed by an exploration strategy. To demonstrate the exploration strategy can be scalable, the same strategy and parameters are applied to robots with two, three and four tubes.

## Methods

As normal in reinforcement learning the inverse kinematics problem is formulated as a MDP where the action, state and reward of the MDP model is detailed as follows.

### MDP formulation

*State*

The state is a combination of the cylindrical representation defined in Eq. 2, the current Cartesian end-effector position *g* and the desired Cartesian end-effector position $\hat{g}$,

6 $$\begin{aligned} s = \left( \gamma _1, \gamma _2, \dots , \gamma _n, g, \hat{g} \right) . \end{aligned}$$

*Action* The action is a change in extension and rotation at one timestep with separate limits for rotation and extension. The rotation limits are set to $\pm ~5^\circ$ and the extension limits are set to ± 0.1 mm. In model-free deep reinforcement learning, the agent can select any value in the continuous range in the limit interval.

7 $$\begin{aligned} a = \left( \varDelta \alpha _1, \varDelta \beta _1, \varDelta \alpha _2, \varDelta \beta _2, \dots , \varDelta \alpha _n, \varDelta \beta _n \right) . \end{aligned}$$

**Table 1:** Concentric tube robot environment parameters

Term	Symbol	Unit	1st tube	2nd tube	3rd tube	4th tube
Curvature	$\kappa$	m$^{-1}$	16.0	9.0	4.0	2.0
Overall length	*L*	mm	150	100	70	20

*Reward* The reward is the scalar value returned by the environment as feedback to the agent from the chosen action at the current timestep. Sparse rewards can be more effective than dense rewards when using hindsight experience replay (HER) for continuous action environments [1]. Moreover, dense rewards are difficult to shape to push the agent towards a desired behaviour. If the error is defined as:

8 $$\begin{aligned} e = \sqrt{(g_x - \hat{g}_x)^2 + (g_y - \hat{g}_y)^2 + (g_z - \hat{g}_z)^2}. \end{aligned}$$

The reward function can then be defined as:

9 $$\begin{aligned} r = \left\{ \begin{array}{ll} 0, &{}\quad e \le \delta \\ -1, &{}\quad {\text {otherwise}}, \end{array} \right. \end{aligned}$$

where $\delta$ is the goal tolerance. The tolerance used in this work is 1 mm during training. An episode consists of a certain number of timesteps for the agent to interact with the environment, before a reset is initiated or the desired goal has been reached. The reward function is calculated at each timestep and is cumulative through the episode, therefore the agent is incentivized to use the fewest timesteps to the achieve desired goal.

*Policy learning* A MLP network is used to model the policy. The network has inputs size that of the environment state dimension and outputs size that of environment action dimension. With a MDP defined, any standard reinforcement learning method that is compatible with continuous state and action spaces can be applied to learn a policy. The chosen method was deep deterministic policy gradient (DDPG) [13]. DDPG outperforms other algorithms in inherently stable environments [10]. Successes in training with DDPG are sparse, it is very unlikely to achieve the desired goal during training in a large workspace. HER was chosen to add successful samples by appending saved failed episode trajectories with future goal sampling strategy with $k=4$ [1].

### Simulation

The kinematics model of the concentric tube robot is the dominant stiffness model [5]. For tube *i*, rotation, $\alpha _i$, is relative to the base of the tube, $\kappa _i$ is the constant curvature and $L_i + \beta _i$ is the extension length. A transformation representing the curvature for a tube is defined as

10 $$\begin{aligned} \mathbf{T} _{curv,i}&= \left[ \begin{matrix} c^2_{\alpha } (c_{\kappa \left( L+\beta \right) } - 1) + 1 &{} s_{\alpha } c_{\alpha } (c_{\kappa \left( L+\beta \right) } - 1) \\ s_{\alpha } c_{\alpha } (c_{\kappa \left( L+\beta \right) } - 1) &{} c^2_{\alpha } (1 - c_{\kappa \left( L+\beta \right) }) + c_{\kappa \left( L+\beta \right) } \\ c_{\alpha } s_{\kappa \left( L+\beta \right) } &{} s_{\alpha } s_{\kappa \left( L+\beta \right) } \\ 0 &{} 0 \end{matrix}\right. \nonumber \\&\qquad \qquad \left. \begin{matrix} - c_\alpha s_{\kappa \left( L+\beta \right) } &{} \frac{L+\beta }{\kappa } c_\alpha (c_{\kappa \left( L+\beta \right) } - 1) \\ - s_\alpha c_{\kappa \left( L+\beta \right) } &{} \frac{1}{\kappa } s_\alpha (c_{\kappa \left( L+\beta \right) } - 1) \\ c_{\kappa \left( L+\beta \right) } &{} \frac{1}{k} s_{\kappa \left( L+\beta \right) } \\ 0 &{} 1 \end{matrix}\right] , \end{aligned}$$

where trigonometric functions $\cos \left( \theta \right)$ and $\sin \left( \theta \right)$ are shown as $c_{\theta }$ and $s_{\theta }$. For the end effector of a robot of *n* tubes, the forward kinematics can be defined as

11 $$\begin{aligned} \mathbf{T} _{ee} = \prod ^{n}_{i} \mathbf{T} _{curv,i} \end{aligned}$$

**Fig. 3:**
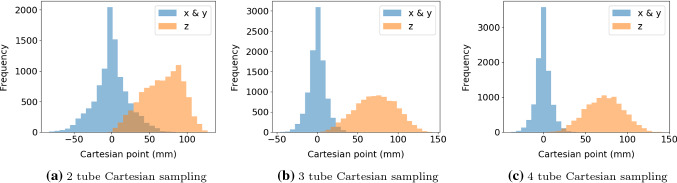
10,000 Cartesian point sampling distribution

*Desired goal sampling* When sampling desired goals from simulation for reinforcement learning, the Cartesian space sampling is not uniform for concentric tube robots with constraints. The desired goals are chosen to be achievable goals by the robot, and therefore, must satisfy the constraints found in Eqs. 4 and 5. There are no such constraints on $\alpha$ and rotation sampling is uniform. For $\beta$, the extensions are constrained therefore a bias in Cartesian desired goal points exists as shown in Fig. 3. To reduce the end effector position stagnating in the biased area, the joint values of the robot are not re-sampled at the end of the episode, only the desired goal is re-sampled.

**Fig. 4:**
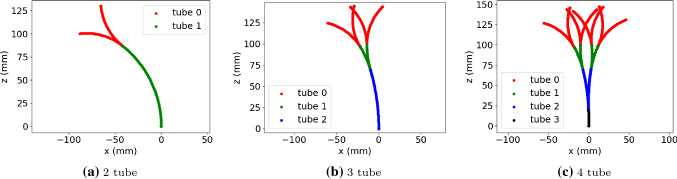
Illustration of robot in full extension with tube rotations 0$^{\circ }$ and 180$^{\circ }$

*Environment and workspace* The tube parameters found in Table 1 define curvatures and overall lengths of each tube and are taken from a previously reported hardware system [8]. With these parameters, and the transform in Eq. 10, the entire workspace of each tube configuration is defined. Figure 4 illustrates the 2, 3 and 4 relative tube lengths and curvatures and the general workspace.

### Exploration

The learned policy, $\mu (s_t | \theta )$, is the MLP network with weights $\theta ^\mu$, state $s_t$, and timestep *t*, will output the next best action, $a_t$. Using an exploration strategy, noise is added to the selected action during training. The three exploration strategies investigated are as follows.

*Zero-mean multivariate Gaussian noise* Given a standard deviation, each action during training is perturbed by sampling a value from a zero-mean Gaussian distribution and arithmetically adding it to the selected action.

12 $$\begin{aligned} a_t = \mu (s_t | \theta ^\mu ) + \mathcal {N} (\varvec{0}, \varvec{\varSigma }) \end{aligned}$$

Often a single standard deviation multivariate Gaussian, such that $\varvec{\varSigma } = \sigma ^2 \varvec{I}$, is used as actions and tend to be of the same units. With a multiple standard deviation multivariate Gaussian, each action index can have an independent standard deviation. For concentric tube robots, extension and rotation joints are of different units therefore independent standard deviations are required. This co-variance matrix is a diagonal matrix with $\sigma _\alpha$ and $\sigma _\beta$ in the index associated with rotation or extension.

*Ornstein–Uhlenbeck noise* Ornstein–Uhlenbeck noise process was the original noise process in the DDPG work [13]. The noise is temporally correlated allowing to set a long-term mean $\varvec{\mu }$. The process moves towards $\varvec{\mu }$ with a given standard deviation $\varvec{\varSigma }$ at a rate $\theta$ and current value $\varvec{x_t}$ over timesteps of the episode and is reset with an episode termination.

13 $$\begin{aligned} a_t = \mu (s_t | \theta ^\mu ) + OU \left( \varvec{x_{t}}, \theta , \varvec{\mu }, \varvec{\varSigma } \right) \end{aligned}$$

We choose to keep rotation noise zero-mean Gaussian, done by setting the initial and long term mean to zero. The standard deviation for rotation noise is the same as for multivariate Gaussian. For extension, we choose to push actions towards extension by setting the initial mean to zero and long term mean to the minimum extension action, as small $\beta$ results in extension. We found $\theta =0.3$ to be appropriate for the length of episode in the environments. The co-variance matrix is similar to multivariate Gaussian noise but a different $\sigma _\beta$.

14 $$\begin{aligned} \begin{aligned} \varvec{\mu } = \left[ \begin{matrix} 0 \quad \texttt {min}(\varDelta \beta _1) \quad \dots \quad 0 \quad \texttt {min}(\varDelta \beta _n) \\ \end{matrix} \right] ^\mathrm{T} \end{aligned} \end{aligned}$$

*Parameter noise* Parameter noise adds noise directly to the policy network weights during training for exploration [18]. Zero mean multivariate Gaussian distribution of size equal to the parameter vector of the policy network is sampled and used to perturb the policy weights directly.

15 $$\begin{aligned} a_t = \mu (s | \theta ^{\mu } + \mathcal {N} (\varvec{0}, {\varvec{\sigma }^2} \varvec{I})) \end{aligned}$$

Adding noise directly to the agent’s parameters allows for more consistent exploration across timesteps, whereas exploration added to actions leads to unpredictable exploration which is not correlated to the agent’s parameters [18].

We investigate these exploration strategies in terms of accuracy and scalability with respect to number of tubes. For each noise type the base hyperparameters of DDPG and HER were found with a full hyperparameter search with 1000 trials, 20,000 episodes per trial, a median pruner and a random sampler. The cost function was negative mean cumulative reward in evaluation. This search was performed on the simplest environment, a single tube environment, due to the large number of hyperparameters in the search to optimize. The results of this search are in Table 2 with cost 50.6. In this search, $\sigma = 0.35$ was found. Next each individual exploration strategy has hyperparameters that need to be tuned. Again, a 1000 trial, 20,000 episodes per trialsearch is performed in a two tube environment with a median pruner and random sampler to tune the hyperparameters of each exploration strategy. The search results were $\sigma _{\alpha } = 0.025$ and $\sigma _{\beta } = 0.00065$ with 60.2 cost for multivariate Gaussian, $\sigma _{\beta } = 0.00021$ with Ornstein–Uhlenbeck with 81.0 and $\sigma = 0.24$ with 41.2 cost for parameter noise. Hyperparameter searches were performed not to find optimal hyperparameters, rather this search is done to prevent learning instability inherent in model-free algorithms [16]. Henceforth we reference type 1, zero mean multivariate Gaussian noise with a single standard deviation, type 2, zero mean multivariate Gaussian noise with multiple standard deviations, type 3, parameter noise and type 4, Ornstein–Uhlenbeck.

**Table 2:** Base hyperparameters table

Hyperparameter	Value
Future sampled goals	4
Buffer size	10000
Batch size	256
Gamma	0.95
Tau	0.001
Random exploration	0.294
Actor and Critic learning rate	0.001
Actor and critic hidden layers	[128, 128, 128]

**Fig. 5:**
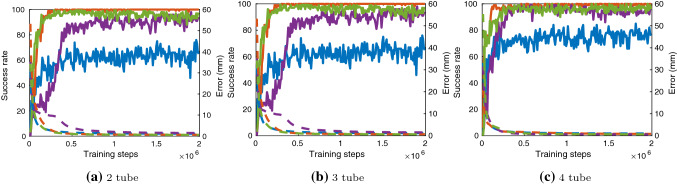
Success rate and evaluation error at training episodes. Solid lines are success rate and dashed lines are error. Blue is type 1, orange type 2, purple type 3 and green type 4

**Fig. 6:**
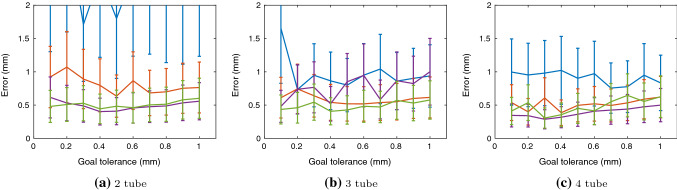
Goal tolerance and resulting mean and standard deviation evaluation error. Blue is type 1, orange type 2, purple type 3 and green type 4

## Experiments and results

For training, we used a server cluster with Intel Xeon Gold 6140 18C 140W 2.3GHz with 19 parallel workers, 2 million timesteps [17] and stable baselines [11]. For each environment, there are four experiments for each exploration strategy for a total of 12 experiments to compare strategies. We also perform additional experiments to demonstrate features of the learned policy. The first additional experiment is evaluating varying the goal tolerance after training. The training goal tolerance was 1.0 mm but errors can be reduced by lowering this goal tolerance in evaluation. Second, we demonstrate following varied trajectories on a *z*-plane with *z*-values at high extension, to test if the joint to Cartesian sampling bias has affected the learned policy.

The results of training shown in Fig. 5 illustrate that all exploration strategies have different convergence. Type 1 noise converges to a success rate of about 60% for 2 and 3 tubes and 70% for 4 tubes while type 2 noise converges to around 95–99% in all tube environments. Type 1 and type 2 noise only differ with how the mean and variance are represented as both are Gaussian noise processes. The main difference between type 1 and type 2 is the separation of standard deviation values for extension and rotation. Separation of these joints in exploration is crucial for convergence. The error side of Fig. 5 show errors reduce incrementally with timesteps and the final evaluation error is shown in Fig. 6 with the goal tolerance at 1.0 mm. With low success rate, high error values for type 1 noise appear in all environments however, high success rate does not necessarily indicate lowest errors. For example, in Fig. 6a, although type 2 performs the best in success rate, it is actually type 3 and 4 that have the lowest errors. This indicates type 2 has only learned the minimal goal tolerance value while type 3 and type 4 have learned further towards the desired goal. Looking from a number of tube scalability perspective, Fig. 5 illustrates with more tubes convergence occurs in less timesteps. Joint to Cartesian sampling bias of desired goal points and redundant joint solutions are the main reasons this behaviour is seen.

**Table 3:** Trajectory following errors with $z=100$ mm for 2 tube and $z=125$ mm for 3 and 4 tube robots

	Circle		Square		Triangle	
	Mean	Std	Mean	Std	Mean	Std
*2-tube*						
Type 1	2.29	1.09	4.35	1.14	3.10	0.80
Type 2	0.82	0.27	1.03	0.48	1.10	0.40
Type 3	0.60	1.21	0.34	0.07	1.19	0.92
Type 4	0.68	0.37	0.31	0.11	0.99	0.91
*3-tube*						
Type 1	4.21	2.31	4.21	3.42	4.21	6.47
Type 2	0.59	0.59	1.84	0.52	0.85	0.19
Type 3	2.55	1.32	2.50	1.26	0.37	0.15
Type 4	0.33	0.12	0.40	0.17	0.59	0.07
*4-tube*						
Type 1	2.27	0.83	2.95	0.86	1.72	1.05
Type 2	1.57	0.98	1.24	0.62	1.22	0.68
Type 3	0.54	0.17	0.39	0.06	0.66	0.40
Type 4	0.32	0.07	1.23	0.69	0.69	0.26

**Fig. 7:**
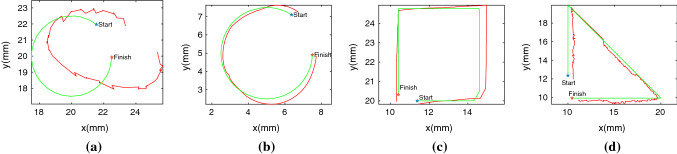
Green is the desired trajectory and red is the followed trajectory. **a** 2 tube type 1, **b** 3 tube type 4, **c** 2 tube type 4 and **d** 4 tube type 3

In our first additional experiment, we vary goal tolerance after training during evaluation shown in Fig. 6. Even though training is done with a 1.0 mm tolerance, if we vary the goal tolerance to 0.4 mm in Fig. 6b, the mean error is at a minimum for type 4. Similar behaviour is seen in the other noise types as well. We believe this indicates the policy learned in some cases, can perform better than the goal tolerance it was originally trained on. We hypothesize this result can be used to vary goal tolerance in a decaying way during training to improve training speed and convergence. Initially, high goal tolerance will allow for quick episodes and success, with subsequent episodes, having a better trained policy, will be more successful in reaching the goals with lower tolerance.

**Fig. 8:**
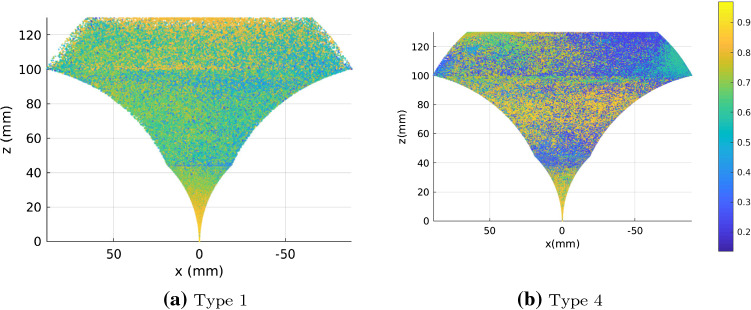
Two tube environment with parameter noise type and 0.6 mm goal tolerance. RGB *Q*-values are normalized between 0 (blue) and 1 (yellow) and visualized

The trajectory following experiments consists of following a circle, square and triangle for each noise type in each environment. The circle will only require rotation actions but the square and triangle will require a combination of rotation and extension actions making them more difficult to follow. In Table 3, we do not see higher errors for square and triangle trajectories in all cases. We hypothsize this is because the joint to Cartesian sampling bias has been overcome in these cases and errors generated are similar for extension and rotation actions. The results from the original experiments are echoed, with type 1 performing poorly as seen in Fig. 7a and other sample trajectories are given in Fig. 7. We found that the location of the shape did play a small role in error results, depending on tube and noise type. We ensure we are in the same quadrant but although rotation exploration is good, it is not equal and requires further study. A more sophisticated controller would also perform better as here we are simply appending the goal in the algorithm and running continuously. To visualize differences between explored and unexplored workspaces, we plot *Q*-value RGB point cloud data of a two tube robot with type 1 noise and type 4 noise at achieved goal points during training in Fig. 8. In Fig. 8a, edges of the workspace at extension show sparse points, indicating there are unvisited sites. Comparatively, in Fig. 8b, the edges are densely packed with points. Looking at the *Q*-value RGB, Fig. 8a, is very homogeneous whereas Fig. 8b is not. We think this is because a poorly approximated critic function will output similar *Q*-values for most state-action pairs since the parameters of the network are sub-optimal. A correctly trained critic will be able to compute varying *Q*-values based on the current state and selected action resulting in a wide range of *Q*-values.

DDA inverse kinematics solver comparisons are difficult to interpret as tube parameters [2] and unmodelled noise processes and perturbations in hardware affect accuracy results and we report results from other work for completeness only. For qualitative comparison, Bergeles et al. [2] report an error of $\sim$ 0.8 mm of extension and $0.1^{\circ }$ rotation for a 3 tube robot with a variable and fixed curvature section in simulation. Grassmann et al. [8] do not record simulation results but presents hardware results of a 3 tube robot of 4.0 mm extension and $8.3^{\circ }$ rotation error. Using type 4 noise on a 3 tube robot in simulation with a dominant stiffness model, our results show an average extension error of 0.44 mm and $0.3^{\circ }$ or $\sim$ 0.5 mm Cartesian error when the desired joint goal and achieved joint goal match due to multiple solutions.

## Conclusions

In this paper, we investigated different noise types to achieve model-free reinforcement learning for control of concentric tube robots in surgical applications. We explored the effect on sampling bias and scalability with respect to the number of degrees of freedom within numerical simulations and demonstrated that reinforcement learning-based DDA is viable for training a dominant stiffness model given correct exploratory noise and hyperparameter selections. We found Ornstein–Uhlenbeck and parameter noise to perform well in environment exploration with multiple tube robots following different trajectory paths to demonstrate the control policy. Interestingly, changing the goal tolerance from training during evaluation can result in lower errors which can be used to improve training complexity and speed as well as convergence in simulation environments. Although fetal surgery is performed manually today, with novel robot designs and potential of concentric tube robots, our model-free inverse kinematics method can aid in future robotic path planning and teleoperation for fetal and other MIS interventions.
